# Successful contact tracing systems for COVID-19 rely on effective quarantine and isolation

**DOI:** 10.1371/journal.pone.0252499

**Published:** 2021-06-03

**Authors:** A. James, M. J. Plank, S. Hendy, R. Binny, A. Lustig, N. Steyn, A. Nesdale, A. Verrall

**Affiliations:** 1 School of Mathematics and Statistics, University of Canterbury, Christchurch, New Zealand; 2 Te Pūnaha Matatini, Auckland, New Zealand; 3 Department of Physics, University of Auckland, Auckland, New Zealand; 4 Manaaki Whenua, Lincoln, New Zealand; 5 Hutt Valley District Health Board, Lower Hutt, New Zealand; 6 Department of Pathology and Molecular Medicine, University of Otago, Dunedin, New Zealand; Swiss Tropical and Public Health Institute, SWITZERLAND

## Abstract

Models of contact tracing often over-simplify the effects of quarantine and isolation on disease transmission. We develop a model that allows us to investigate the importance of these factors in reducing the effective reproduction number. We show that the reduction in onward transmission during quarantine and isolation has a bigger effect than tracing coverage on the reproduction number. We also show that intuitively reasonable contact tracing performance indicators, such as the proportion of contacts quarantined before symptom onset, are often not well correlated with the reproduction number. We conclude that provision of support systems to enable people to quarantine and isolate effectively is crucial to the success of contact tracing.

## Introduction

The WHO guidelines for control of COVID-19 emphasise three crucial components of an effective strategy: test, trace, and isolate [1]. Collectively, this system of rapid case and contact management has become one of the key public health tools in the fight against COVID-19 worldwide [2–4]. Contact tracing has been crucial in controlling several disease outbreaks, notably SARS, MERS and Ebola [5, 6]. While contact tracing alone is unlikely to contain the spread of COVID-19 [7, 8], it may allow population-wide social distancing measures to be relaxed. There is a need for robust ways to measure the effectiveness of contact tracing in reducing the spread of COVID-19. The effective reproduction number, *R*_*eff*_, measures the current transmission rate of the virus and the aim of many public health interventions is to reduce *R*_*eff*_. However, it is difficult to disentangle the effects of multiple simultaneous interventions, such as case-isolation, contact tracing, and population-wide restrictions, using real-time estimates of *R*_*eff*_ alone [9]. We therefore need reliable operational indicators to measure the impact of contact tracing [10].

Quarantine refers to the separation of individuals who may have been exposed to the virus but are currently pre-symptomatic or asymptomatic, and is distinct from isolation of symptomatic or confirmed cases [11]. In reality, the quarantining of contacts and isolation of cases are complex processes and their effectiveness depends on a range of factors. Complete isolation of all confirmed cases is impractical in most countries: some contact with household members and essential service providers or healthcare workers is inevitable in at least some cases. Barriers to effective isolation are higher in communities with high levels of socioeconomic deprivation, insecure employment, and limited entitlement to paid sick leave. Quarantine of pre-symptomatic or asymptomatic contacts, the majority of whom are likely not infected, is even more challenging [12] and taking time off work when not symptomatic is likely to be impossible for many. In countries with large outbreaks, this could affect tens of thousands of people at any given time and require closure of entire workplaces for extended periods. As digital proximity-based contact tracing systems are introduced, the number of false positives could increase further. This suggests that quarantine is likely to involve precautionary measures rather than complete isolation and therefore to be less effective than isolation of confirmed cases in reducing onward transmission.

The contribution of contact tracing to reductions in transmission is difficult to quantify in real-time because of incomplete and lagged data, and the difficulty of disentangling the effects of multiple interventions and behavioural changes over time. Mathematical modelling is useful because it provides a rigorous framework in which to estimate the effect of contact tracing under different assumptions about the key properties of the system, such as speed, coverage and outcomes. Models have been used to investigate various aspects and determinants of contact tracing for COVID-19, for example: heterogeneous contact networks [13], different quarantine periods and test-to-release settings [14]; digital contact tracing methods [2, 15], and speed and coverage of contact tracing [7, 8, 16]). Most existing models assume that quarantine or isolation completely prevents further onward transmission [2, 13, 16, 17], or assume fixed values for the proportion of transmission prevented by quarantine and isolation [8, 14]. Other models assume the effectiveness of isolation and the probability of being traced are interchangeable, i.e. a system where 50% of contacts are traced and isolation is 100% effective is assumed to have the same outcomes as one where 100% of contacts are traced and isolation reduces onward transmission by 50% [7]. Endo [18] investigated the potential of contact tracing backwards as well as forwards (i.e. tracing the source of infection, as well as potential secondary cases) to reduce transmission, using a model that allowed for different levels of effectiveness of quarantine. However, they did not explicitly investigate the importance of quarantine effectiveness relative to other parameters, nor did they consider the problem of how to estimate the effectiveness of quarantine in the absence of direct data.

**Fig 1:**
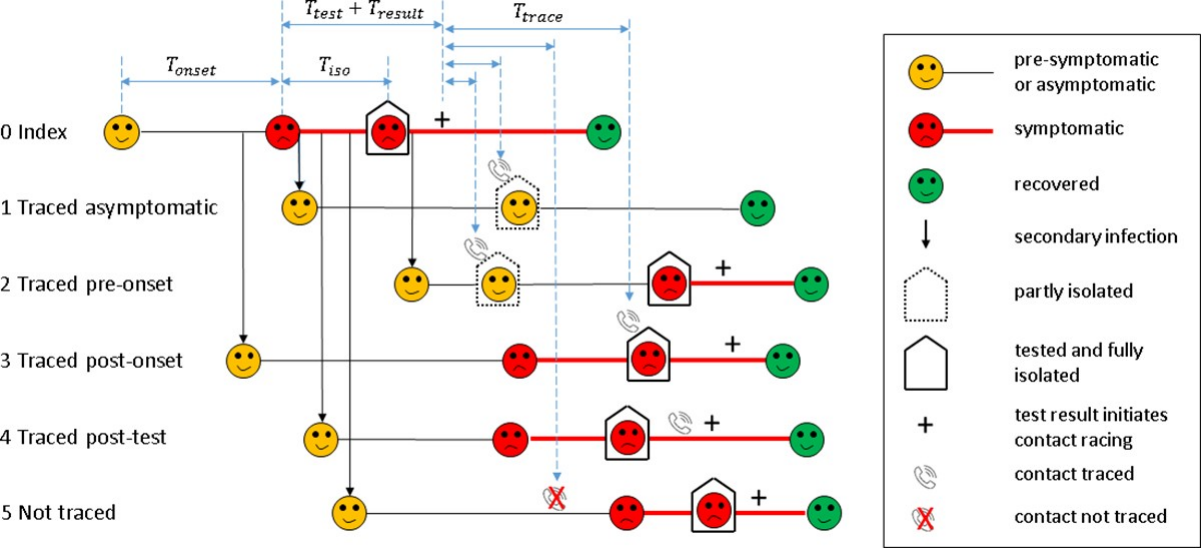
Schematic diagram of the contact tracing model. Infected individuals are initially not symptomatic (yellow). Some infections eventually develop symptoms (red), others remain asymptomatic for the duration of infection. For the index case who was not traced (0), there is a delay between onset of symptoms (red) and getting tested. Isolation occurs at some point between symptom onset and testing. There is a subsequent delay from testing to the test result being returned (+) and tracing of contacts. Traced contacts (1–4) are quarantined when contacted by public health officials (phone icons) and are isolated and tested immediately on symptom onset. Traced contacts (3) who are already symptomatic prior to being traced are isolated immediately when contacted. Traced contacts (4) that have already isolated prior to being traced are not affected. Contacts that cannot be traced (5) may still get tested and isolated after developing symptoms, but this is likely to take longer. Asymptomatic infections (1) do not get tested or isolated, but will be quarantined if they are a traced contact. Some asymptomatic infections may be untraced, in which case they will not be quarantined or isolated (not shown).

We explicitly model the effectiveness of contact quarantine and case isolation as independent variables, and allow quarantine of contacts who are currently not symptomatic to be less effective than isolation of confirmed cases (Fig 1). This is a realistic model assumption that reflects greater likelihood of behaviour change, increased levels of support, or greater mandatory regulation for isolation of cases following a positive test result. This allows us to separately investigate the impact of increasing effectiveness of quarantine and isolation. We use a model calibrated using data on time from symptom onset to isolation and test result of COVID-19 cases in New Zealand, where community transmission of the virus was eliminated in June 2020 [19].

Measuring the input parameters for the contact tracing model empirically is not straightforward. We therefore evaluate four potential performance indicators for the impact of the contact tracing system. We show that seemingly straightforward indicators, such as the proportion of cases quarantined before symptom onset, can be misleading. We propose a new indicator based on the time between quarantine or isolation of an index case and quarantine or isolation of secondary cases and show that this is a more reliable measure of the reduction in *R*_*eff*_. Our results highlight the importance of establishing support systems to enable individuals to quarantine and isolate effectively [20]. They also demonstrate that effective contact tracing requires a skilled, professional workforce that can trace downstream contacts of a positive case, as well as upstream contacts to determine the source of infection and provide the high-quality data needed.

## Methods

### Model

We use a continuous-time, age-structured branching process model [21] for the transmission of COVID-19 in the presence of contact tracing and case isolation (Fig 1)–see S1 File for details. Individual infectiousness is modelled using a time-dependent infection kernel. Infections are split into two categories: those who eventually develop symptoms and those who remain asymptomatic for the whole infectious period. The asymptomatic fraction decreases with age. For cases who eventually develop symptoms, the infection kernel is shifted according to the time of symptom onset, such that 35% of onward transmission occurs during the pre-symptomatic phase [22, 23]. Asymptomatic cases follow the same shape of infection kernel as eventually-symptomatic cases, but are only 50% as infectious.

The key input parameters for the contact tracing model are: (i) the proportion of contacts successfully traced; (ii) the mean time taken to trace contacts following a positive test result for the index case; and (iii) the effectiveness of contact quarantine and case isolation in reducing onward transmission. The time for tracing ranges from zero, which can usually only be achieved for household contacts or via a highly effective digital tracing system, through to a mean of 6 days. When the average tracing time is longer than 6 days, the results showed that tracing made very little difference as most secondary cases self-identified prior to being traced. Traced contacts of a positive case who are not currently symptomatic go into quarantine. Upon symptom onset, traced contacts go into isolation and are tested. We assume that untraced symptomatic cases are also tested and isolated, but that there is a delay from onset to isolation and testing (see S1 File for distributions). The isolation and testing model is calibrated using data on time from symptom onset to isolation and test result in cases of COVID-19 in New Zealand in April to May 2020. The delay between testing (when isolation starts) and test result being returned (when tracing starts) in this dataset was 1 day on average. However, the shape of the assumed distribution for tracing time means the model can still be applied to countries where testing is slower by increasing the mean time from testing of the index case to tracing of contacts. We assume that all symptomatic individuals eventually get tested.

We run the model for three tracing probabilities *p*_*trace*_, 0% (no contact tracing), 50% and 100% (all contacts traced), and a range of tracing speeds from instant tracing (${\bar{T}}_{trace}=0$) to a mean tracing time of ${\bar{T}}_{trace}=6$ days. Isolation and quarantine are always between 50% and 100% effective, i.e. transmission is either halved or reduced to zero during this period. We assume that isolation of symptomatic cases is always at least as effective as quarantine of pre-symptomatic or asymptomatic contacts. We measure the impact of contact tracing via the reduction in the effective reproduction number *R*_*eff*_, defined as the mean number of secondary infections per case.

To analyse the sensitivity of model results to testing and tracing parameter values, we run the model with combinations of parameters randomly and independently generated from uniform distributions on the following intervals: tracing probability *p*_*trace*_∈[0,1], mean tracing time ${\bar{T}}_{trace}\in [0,5]$, quarantine effectiveness between 50% and 100%, isolation effectiveness between quarantine effectiveness and 100%, testing probability for untraced clinical cases *p*_*detect*_∈[0.3,1] and reduction in transmission due to social distancing measures (0%−50%). We also test the sensitivity of the model to the proportion of transmission that occurs before symptom onset, *p*_*pre*_, by running simulations for *p*_*pre*_ = 20%, 35% (used in the main analysis) and 50%.

### Data

We used the New Zealand EpiSurv dataset, centrally managed by Environmental Science and Research (ESR) on behalf of the Ministry of Health (accessed 6 August 2020)–see S1 File for details. We included the N = 101 confirmed and probable cases with a symptom onset date between 8 April and 8 May 2020 and exclude those with a recent international travel history (N = 8). All 93 included cases eventually developed symptoms and had a symptom onset and either a quarantine or isolation date recorded.

We calculated each of the following four performance indicators across all cases in the sample.

- Proportion of secondary cases quarantined or isolated within 4 days of quarantine or isolation of the index case.
- Proportion of secondary cases quarantined or isolated within 4 days of symptom onset in the index case.
- Proportion of secondary cases with symptom onset within 4 days of onset in the index case (i.e. serial interval less than 4 days).
- Proportion of cases quarantined before symptom onset.

Quarantine refers to limiting contacts of asymptomatic or pre-symptomatic contacts, while isolation refers to symptomatic cases. Some cases have only a quarantine time (e.g. case 1 in Fig 1), some have only an isolation time (cases 3–5 in Fig 1), and some have a quarantine time and a later isolation time (case 2 in Fig 1). For simplicity, the indicators above are defined using the quarantine time if it exists, or the isolation time if not. Indicators (B)-(D) have been suggested by [10] as potential indicators of the effectiveness of a contact tracing system.

Indicator (D) can be calculated for all *N* = 93 cases in the dataset (vertical dashed line in Fig 3D). Indicators (A)-(C) require an index case to be identified, which means they can only be calculated for 81 cases. Of these 81 cases, 37 had multiple potential index cases identified. For these cases, the value of each indicator was calculated twice: once by selecting the index case corresponding to the smallest value of the indicator; once by selecting the index case corresponding to the largest value of the indicator. This gives a range of possible values of the indicator for the New Zealand dataset, indicated by the two vertical dashed lines in Fig 3A–3C.

## Results and discussion

### Factors determining the impact of contact tracing

**Fig 2:**
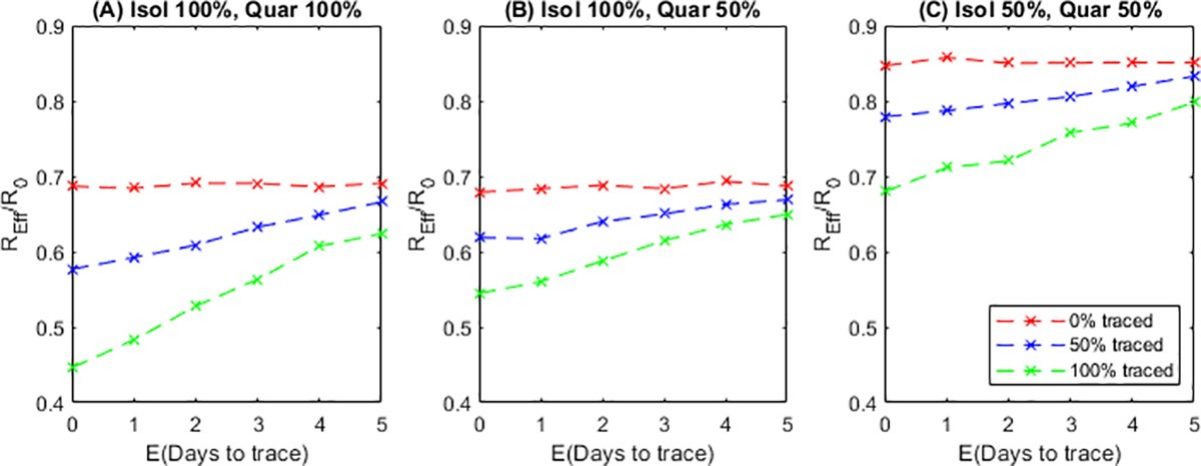
The impact of contact tracing on the effective reproduction number *R*_*eff*_ is strongly affected by the proportion of contacts traced, the tracing speed and the effectiveness of quarantine and isolation. Graphs show the effective reproduction number relative to the no-control scenario (*R*_*eff*_/*R*_0_) against the mean tracing time, *E*(Days to trace), for a range of tracing probabilities and quarantine/isolation effectiveness. *R*_*eff*_/*R*_0_ = 1 when there is no contact tracing or case isolation.

Under a case isolation policy with no contact tracing (Fig 2, red lines), more effective isolation leads to a decrease in the effective reproduction number *R*_*eff*_ relative to the no-control scenario (compare Fig 2A, 2D and 2F, red lines). Across all scenarios, fast tracing results in lower *R*_*eff*_ than slow tracing (Fig 2). If tracing is fast, then tracing more contacts reduces *R*_*eff*_ further; if tracing is very slow (mean tracing time > 5 days), the system is ineffective regardless of the proportion of contacts traced and *R*_*eff*_ is similar to the no-tracing scenario. In Fig 2, we fixed the mean time from testing to the test result being returned and varied the mean time from the return of a positive test to the tracing of contacts. The results are similar if the mean tracing time is fixed and the mean testing time varied: the most important parameter is the total time from testing the index case to tracing their contacts.

In reality, the tracing speed and coverage are likely to be inter-dependent: tracing a few contacts, particularly household or other close contacts, can be done quickly; but tracing a higher proportion of all contacts is likely to include some who take longer to trace, so the mean tracing time will increase. Our results show if the mean tracing time is more than 6 days, there is little benefit in trying to trace more contacts and the priority should be faster tracing of the easier-to-trace contacts.

The effectiveness of quarantine and isolation is a crucial determinant of the impact of contact tracing on *R*_*eff*_. Because there is significant pre-symptomatic transmission of COVID-19 [22, 23], fast tracing in conjunction with effective quarantine of contacts before symptom onset can greatly reduce spread. Although some countries, notably China, required institutional isolation and monitored quarantine [30], most countries in Europe, North America and Australasia rely on home quarantine for contacts and home isolation for mild cases. Asking individuals to quarantine or isolate but then failing to support them to do so, means, for example, they will either need to go shopping for food and other essential items or have them delivered by family or friends who potentially should also be in isolation. Many individuals may be ineligible for paid sick leave, especially when not symptomatic. Crowded or unsuitable housing may mean isolation is not feasible. Precarious employment situations could be exacerbated by prolonged and possibly repeated absences. Given these realities, it is unlikely that 100% effective isolation and quarantine (Fig 2A) is achievable.

We suggest that a more realistic scenario is one where quarantine only reduces onward transmission by 50% (Fig 2B). If tracing is instantaneous, this provides the same reduction in *R*_*eff*_ as a mean tracing time of 2–3 days with 100% effective quarantine. If isolation of cases is only 50% effective (Fig 2C), improving isolation may be more impactful than faster or more complete contact tracing (Fig 2A, red line). Tracing 50% of contacts with 100% quarantine and isolation effectiveness (Fig 2A, blue line) is substantially better than tracing 100% of contacts with 50% isolation and quarantine effectiveness (Fig 2F, green line). This slightly counterintuitive result is because, regardless of tracing, all individuals who develop symptoms will eventually be tested and isolate. When isolation is very good this gives a better reduction in transmission in comparison to all individuals being traced early but not isolating fully. Many previous models of contact tracing [7] do not distinguish these two scenarios as they assume isolation is 100% effective. There may not necessarily be a trade-off between these two alternatives; the comparison illustrates the relative importance of tracing coverage and effectiveness of quarantine and isolation. Nonetheless, there could be a significant operational trade-off in practice, if contact tracing staff are also involved in providing personalised follow-up to encourage adherence or ongoing monitoring of possible symptoms.

In Fig 2, we have only showed the mean value of *R*_*eff*_/*R*_0_ for each scenario. As these results are obtained from simulations, there is effectively no confidence interval associated with them as the confidence interval can be made arbitrarily narrow by increasing the number of simulations. As individual reproduction numbers are heavy-tailed, e.g. usually at least 50% of individuals infect no secondary cases, the inter-quartile range of individual reproduction numbers is not a helpful measure either. There is however a range of values that would be seen in any individual outbreak. For small outbreaks the variation in *R*_*eff*_ would be very large as there would only be a few cases to consider, when outbreaks are much larger we would expect the value of *R*_*eff*_ to converge to the mean value shown here. Finally, the epidemiological parameters defining the model for an uncontrolled epidemic are kept the same across all scenarios. This means that *R*_0_ is fixed so *R*_*eff*_/*R*_0_ is directly proportional to the absolute mean number of secondary infections caused by an infectious individual.

### Measuring the impact of contact tracing

It is difficult to measure the impact of contact tracing on *R*_*eff*_ directly because other interventions will typically be in place simultaneously. There is also a time lag between implementation and any reduction in *R*_*eff*_ as seen in reported new cases. It is difficult to obtain reliable data to estimate the input parameters for the contact tracing model directly. The mean tracing time is the easiest input parameter to measure, although this could be underestimated if some contacts or cases present to healthcare before being traced. Officially reported values of tracing coverage typically represent the proportion of known contacts traced, neglecting contacts who could not be recalled by the case. To quantify the effectiveness of quarantine and isolation would require detailed information about the number of secondary infections during these periods, which would involve intensive follow-up with quarantined and isolated individuals. Unless this was done for all cases, this intervention itself could bias the sample towards individuals who were isolating more effectively.

**Fig 3:**
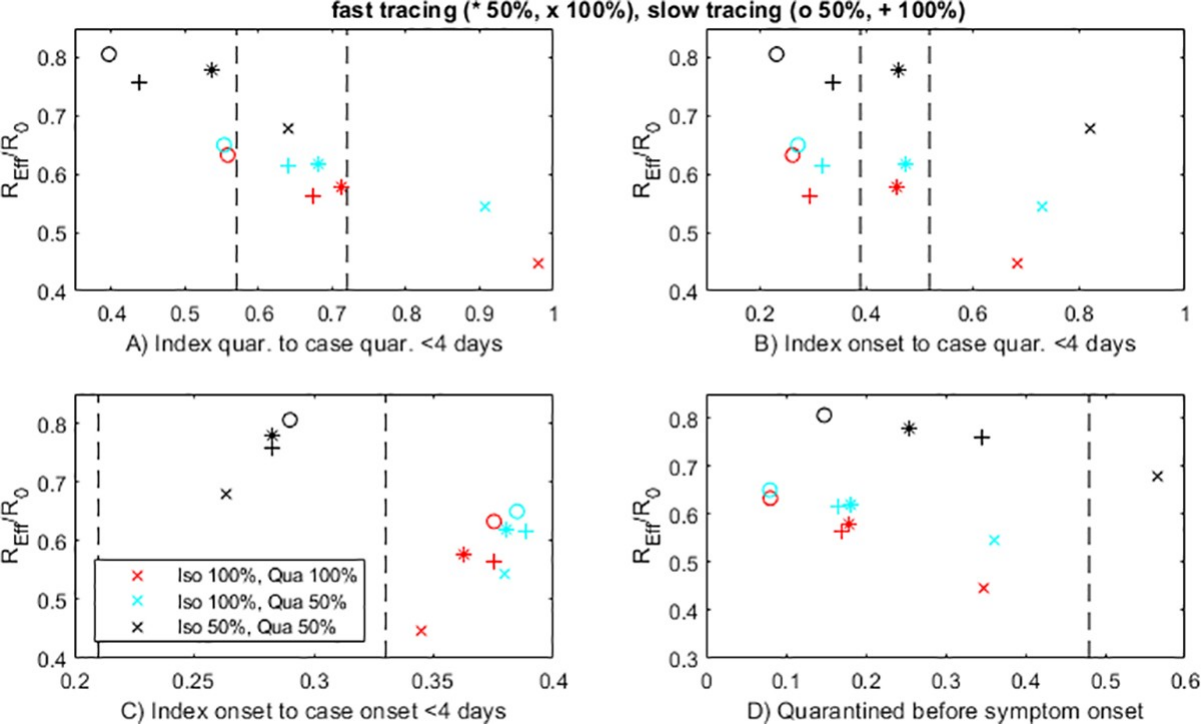
The proportion of cases quarantined or isolated within 4 days of the index case being quarantined or isolated (A) is the most robust indicator of the performance of the contact tracing system, measured by the reduction in effective reproduction number *R*_*eff*_ relative to the no control case. Other indicators (B)-(D) are not reliably correlated with *R*_*eff*_ across all model parameters. Each plotted point corresponds to one combination of model parameters: fast tracing (same time as test result) of 50% of contacts (stars); fast tracing of 100% of contacts (crosses); slow tracing (mean 3 days after test result) of 50% of contacts (circles); slow tracing of 100% of contacts (pluses); varying effectiveness of isolation and quarantine are shown by different colours (see graph legend). Other parameter values shown in Table 1. The horizontal axes show the proportion of cases meeting the specified performance indicator. Vertical dashed lines show the minimum and maximum values of the indicators computed from New Zealand case data. Indicator (D) can be calculated for all *N* = 93 cases in the dataset. Indicators (A)-(C) require an index case to be identified, which means they can only be calculated for 81 cases. 37 of these 81 cases have multiple potential index cases. The left-hand dashed line in (A)-(C) shows the result of selecting the index case corresponding to the smallest value of the indicator; the right-hand dashed line in (A)-(C) shows the result of selecting the index case corresponding to the largest value of the indicator.

There is therefore a need for performance indicators that can be used to estimate the impact of contact tracing on *R*_*eff*_, separately from other interventions and in the presence of uncertainty in parameter values. A robust performance indicator should: (i) be measurable from data routinely collected by public health organisations; and (ii) be closely correlated with *R*_*eff*_ across a broad range of model inputs. Fig 3 shows the relationship between *R*_*eff*_ and each of the four performance indicators (A)–(D) described in Methods. Each point in Fig 3 corresponds to a different combination of the four contact tracing parameters: tracing probability, tracing speed, quarantine effectiveness, and isolation effectiveness. We assume these parameters cannot be measured directly in practice and it is therefore *a priori* unknown which parameter combination applies. To be a reliable measure of the impact of contact tracing, an indicator should be tightly correlated with *R*_*eff*_ across the spectrum of parameter combinations.

**Table 1:** The parameters used in the model and their source.

Parameter	Value	Source
Distribution of exposure to onset (days)	*T*_*onset*_~Γ(*shape* = 5.8, *scale* = 0.95)	Lauer, Grantz [24]
Distribution of generation times (days)	*T*_*s*_−*T*_*onset*_+*t*_*p*_ ~*Weibull*(*shape* = 2.83, *scale* = 5.67)	Ferretti, Wymant [2]
Distribution of onset to testing (untraced contacts) (days)	*T*_*test*_~Γ(*shape* = 1.22, *scale* = 2.17)	NZ Case data
Distribution of onset to isolation (days)	*T*_*iso*_~Γ(shape = 0.62, scale = 3.47)	NZ Case data
Distribution of testing to test result (days)	*T*_*result*_−0.5~*Exp*(0.5)	NZ Case data
Distribution of test result to contact tracing (days)	$T_{trace}∼\Gamma (shape=\frac{\bar{T_{trace}}}{0.1},scale=0.1)$	See scenario parameters
Proportion of secondary infections occurring before symptom onset (in the absence of case-targeted control)	*p*_*pre*_ = 35%	Hellewell, Abbott [7]
Relative infectiousness after quarantine	*c*_*quar*_	See scenario parameters
Relative infectiousness after full isolation	*c*_*iso*_	See scenario parameters
Proportion of symptomatic case who get tested	*p*_*test*_ = 1	Assumed. Varied in sensitivity analysis
Proportion of contacts traced	*p*_*trace*_	See scenario parameters
Age-structured scenario parameters children (0–19 years), adults (19–65 years), elderly (over 65)		
Reproduction number for eventually-symptomatic infections (without case isolation or control)	*R*_*clin*_ = 4.6, 3, 1.3	Estimated from age-adjusted contact rates Compass Research Centre [25]
Relative infectiousness of asymptomatic infections	$R_{sub}^{G}/R_{clin}^{G}=50\%$	Davies, Kucharski [26] Chau, Lam [27]
Proportion of asymptomatic infections	*p*_*sub*_ = 0.8, 0.33, 0.2	Davies, Klepac [28]
Contact probabilities between groups	$\Lambda =\begin{array}{ccc} 0.6 & 0.325 & 0.075\\ 0.1 & 0.825 & 0.075\\ 0.1 & 0.325 & 0.575 \end{array}$	James, Plank [21], Prem, Cook [29]

Indicator (A), the proportion of secondary cases quarantined or isolated within 4 days of quarantine or isolation of the index case, was well correlated with *R*_*eff*_ across variations in all three input parameters. This means that improvements in contact tracing and isolation are reliably associated with improvements in the value of the indicator (Fig 3A). In contrast, indicators (B)-(D), which use time of symptom onset, can be misleading. The reasons for this are subtle and counterintuitive. Indicator (B) depends almost exclusively on tracing speed and proportion of contacts traced, and is insensitive to the effectiveness of quarantine or isolation. If the effectiveness of quarantine and case isolation can be evaluated independently or assured in some other way, this could be a useful indicator, but without this it is not reliable. Indicator (C), which is a function of the realised serial interval, is most sensitive to the effectiveness of quarantine: more effective quarantine produces a shorter mean serial interval by preventing onward transmission late in the infectious phase [31]. An improvement in either the tracing speed or the proportion of contacts traced leads to an apparent deterioration in the value of this indicator. The reason for this counterintuitive result is that, as the contact tracing system becomes more effective in preventing transmission, the remaining untraced cases are dominated by those infected by asymptomatic carriers. These cases cannot be traced in the model, as their index case is undetected, and so continue to spread the virus relatively late in their infectious periods, leading to longer serial intervals. Indicator (D) is the easiest to measure as it can be calculated for all cases, including those that are not associated with a specific index case. However, it is the worst of the four indicators tested, showing apparently poorer outcomes as the effectiveness of quarantine or isolation improves (Fig 3D). This happens because effective case-targeted interventions tend to prevent transmission late in the infectious phase (e.g. case 2 in Fig 1). The remaining cases are skewed towards those that were infected early in the infectious phase of the index case (e.g. case 3 in Fig 1), which are the hardest to trace before symptom onset. Because the indicator can only be calculated for the cases that do eventuate and not those that were prevented, it appears to show that contact tracing is getting worse when it is actually improving.

Fig 3 shows results for two different tracing speeds (instantaneous tracing and mean 3 days from test result to tracing). In some countries, tracing may take longer than 3 days and this means the reduction in *R*_*eff*_ is not as good. We tested mean tracing times up to 10 days and found that longer tracing times were reliably associated with decreases in the value of the indicator (A), but were poorly correlated with values of the other indicators (B)-(D).

The recommended indicator (A), the proportion of cases quarantined or isolated with four days of quarantine or isolation of the index case, is between 56% and 72% for the New Zealand data (Fig 3A, dashed lines)–see Methods. The model results that lie in this range imply that, with no knowledge of the other system parameters, contact tracing and case isolation reduced *R*_*eff*_ by 30–45% during this period. This estimate is subject to substantial noise in the data due to self-reporting of isolation dates and symptom onset dates and uncertainty in assigning index cases. The other indicators give a much more uncertain range for the reduction in *R*_*eff*_.

**Fig 4:**
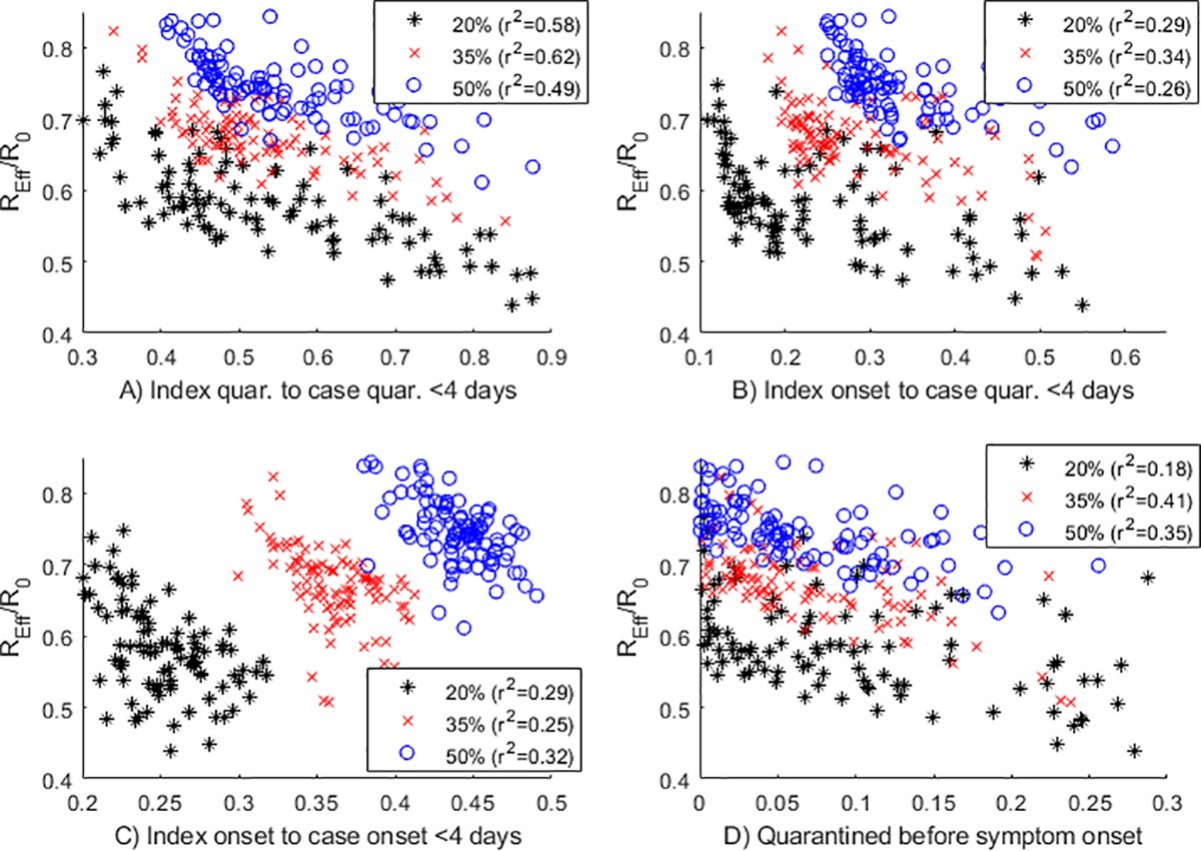
The proportion of cases quarantined or isolated within 4 days of the index case being quarantined or isolated (A) is still the most robust indicator of the performance of the contact tracing system under different levels of presymptomatic transmission (20%, black ‘*’, 35% red ‘x’, 50% blue ‘o’). Other indicators (B)-(D) are not reliably correlated with *R*_*eff*_ across all model parameters. Each plotted point corresponds to a randomly chosen combination of: tracing coverage *p*_*trace*_, tracing speed ${\bar{T}}_{trace}$, quarantine effectiveness *c*_*quar*_, isolation effectiveness *c*_*iso*_, and probability of testing for untraced clinical cases *p*_*test*_. Other parameter values shown in Table 1. The horizontal axes show the proportion of cases meeting the specified performance indicator.

To test the sensitivity of the four performance indicators to model parameters, we ran additional sets of simulations for three different values of the proportion of transmission that occurs before symptom onset (*p*_*pre*_ = 20%, 35%, 50%) and for randomly selected combinations of the tracing coverage *p*_*trace*_ and speed ${\bar{T}}_{trace}$, quarantine and isolation effectiveness *c*_*quar*_ and *c*_*iso*_, probability of testing for untraced clinical cases *p*_*test*_ and level of social distancing (Fig 4). For any given value of the parameter *p*_*pre*_, the recommended indicator (A) has the best correlation (highest *r*^2^) with the reduction in *R*_*eff*_ compared to no contact tracing across different combinations of testing and tracing parameters. Although the exact value of *p*_*pre*_ may be unknown, these results show that provided this parameter is fixed, the recommended indicator (A) gives the most reliable way to estimate the impact of the contact tracing system on reducing transmission.

Additional sensitivity analysis could be run on the proportion of infections which are asymptomatic, fixed in this study at 33%. Changing this parameter would have a quantitative effect on both *R*_*eff*_ and *R*_0_ because of the assumption that asymptomatic infections have a lower transmission rate. It would also influence the effectiveness of contact tracing because it is assumed that asymptomatic infections can be quarantined but not isolated, and any secondary infections from an asymptomatic transmitter cannot be traced. However, the qualitative change in the results would be minimal: the sample size for indicators (B)-(D) would be reduced as these only apply to symptomatic infections as they require a symptom onset date; and the preferred indicator (A) would remain unchanged as it only applies to the quarantine dates of an index-secondary case pair.

## Conclusions

Our results show that a high-quality, rapid contact tracing system, combined with strong support for people in quarantine or isolation, can be highly effective in reducing the spread of COVID-19. In the best cases, it may reduce the effective reproduction number *R*_*eff*_ by up to 60%. In the absence of any control interventions, the basic reproduction number *R*_0_ for COVID-19 is estimated to be between 2 and 4 [32–34]. Containing the spread of COVID-19 requires *R*_*eff*_<1, which implies that moderate social distancing will also be required to control outbreaks. If case isolation or contact quarantine are imperfect, or some contacts are not traced or traced more slowly, then the reduction in *R*_*eff*_ is only around 40%, meaning that stronger social distancing would be required.

In countries where testing is very slow or contact tracing capacity is insufficient, short-term gains in controlling the virus may be made by measures that improve effectiveness of case isolation. This does not diminish the importance of expanding rapid testing and building contact tracing capacity, but recognises that this may take longer to achieve. Case isolation alone is unlikely to contain an epidemic because chains of transmission from infected contacts will continue to grow [8]. Our model assumed that all symptomatic cases were eventually diagnosed, which relies on widespread availability and uptake of testing. Case-targeted measures will be less effective if there is significant case under ascertainment.

We recommend using the time from quarantine or isolation of the index case to quarantine or isolation of secondary cases as a performance indicator for the contact tracing system. If at least 80% of cases are quarantined or isolated within 4 days of quarantining or isolating the index case, this indicates a reduction in *R*_*eff*_ of at least 40%. O’Dowd [35] and Verrall [10] identified key criteria against which to evaluate the contact tracing system, for example that at least 80% of contacts must be quarantined within 4 days of symptom onset in the index case. We have shown that if the effectiveness of quarantine and isolation can be guaranteed, this criterion can be useful to benchmark a system. However, when these are not accurately known, the indicator recommended above is more robust. Case-targeted interventions tend to prevent onward transmission late in the infectious period. This skews remaining cases towards those infected in the pre-symptomatic or early symptomatic phase of the index case, which are more difficult to trace in a timely way. Our work shows that in order to establish the effectiveness of a system, index-case pairs must be determined as accurately as possible, even if this is done *post hoc* with case investigation. Without this information, there is a danger that seemingly simple criteria, such as the proportion of cases quarantined before symptom onset, could give misleading indications of system performance.

The New Zealand contact tracing system is well-established and run by highly trained public health staff. Their work has been critical to the success of New Zealand’s elimination strategy for COVID-19 [36]. However, even this well-established system suffers from noisy data and the difficulties of establishing index-case pairs. Our results show that this system, despite its contribution to eliminating the virus in New Zealand, can still be improved, in particular by reducing the time taken to trace contacts. The dataset we have used comes from a period when New Zealand had strict stay-at-home orders in force, greatly reducing the average number of contacts needing to be traced. An outbreak during a period without stringent population-wide restrictions would place a much greater strain on the contact tracing system.

Our model assumed that a fixed fraction of infections in each age group are asymptomatic [27] and, in the absence of any case-targeted interventions, 35% of all onward transmission from eventually-symptomatic cases occurs during the pre-symptomatic phase. There is a range of empirically derived estimates for these parameters for COVID-19 [2, 22, 23, 37, 38], and the effectiveness of contact tracing is sensitive to these [7]. If the true rates of asymptomatic and pre-symptomatic transmission are less than these assumed values, it is easier to trace a greater number of contacts prior to them becoming infectious, likely making contact tracing more effective. If the true values are higher, it is likely that contact tracing will need to be combined with stronger social distancing measures to contain COVID-19. Nevertheless, our conclusions about robust performance indicators still hold even if the values of these parameters are unknown, provided they do not change over time. Our model does not distinguish between the different types of contact, such as household, work or casual, and each of these groups may experience different speeds to trace and possess different abilities to isolate effectively. These features would be a useful addition to the model but would not undermine the conclusions on isolation effectiveness and robust indicators.

Improving the speed and capacity of contact tracing systems is likely to be more cost-effective than prolonged population-wide social distancing measures, although some level of social distancing may be also be needed to contain a resurgence. The crucial importance of effective quarantine and isolation makes it essential that there is universal provision of social security such as paid leave entitlements for pre-symptomatic or asymptomatic individuals in quarantine, and adequate job security and unemployment benefits. Likewise, the need to rapidly trace the majority of contacts mean that investment in skilled professionals and workers trained in public health work is essential. Experience from the New Zealand contact tracing effort shows that the development of trusted relationships by public health officials is critical to an effective system.

Overreliance on automated contact tracing solutions or the use of contact tracing staff who are not trained in public health work may lead to less favourable outcomes. This does not diminish the importance of developing scalable contact tracing systems, including digitally supported and automated systems. These are an increasingly essential part of the public health response during a major outbreak where public health agencies do not have capacity to manually trace all contacts of new cases. However, it highlights the need to design digital systems that are well integrated with public health agencies, so that the effectiveness of quarantine and isolation is not compromised. Ideally this will allow a smooth transition from digital support of manual contact tracing when case numbers are low, to increasing levels of automation needed if case numbers grow.

## Supporting information

S1 File (DOCX)

S1 Fig Distribution of generation times (time from infection of the index case to infection of secondary cases) with no contact tracing or case isolation. Three published generation time distributions are shown for comparison [2, 39, 40].
(TIF)
