# Supplementary material for: Successful contact tracing systems for COVID-19 rely on effective quarantine and isolation
Source: PLoS One. 2021 Jun 3;16(6):e0252499. doi: 10.1371/journal.pone.0252499 (PMC8174731; doi:10.1371/journal.pone.0252499)
Supplement: S1 File — (DOCX) [file pone.0252499.s001.docx]

**Contents**

Materials and Methods: Model

Model assumptions

Stochastic branching process model

Performance indicators

Data

Empirical calculation of performance indicators

**Materials and Methods: Model**

We use an age-structured continuous-time branching process model [1] to study the transmission of COVID-19 in the presence of contact tracing and case isolation.

**Model assumptions.** The key assumptions of the model are:

- Infected individuals fall into one of two categories: those that eventually develop symptoms and those that remain asymptomatic for the entire infectious period. Asymptomatic infections are assumed to have a reduced infectiousness (see Table 1 for parameter values) and to remain untested
- For eventually-symptomatic infections, the time between exposure and onset of symptoms (in days) $T_{onset}$is drawn from a gamma distribution

$$T_{onset} \sim\Gamma\left( 5.8, 0.95 \right).$$

- The times of secondary infections $T_{s}$ are drawn from an individual-specific generation time distribution, which is shifted according to the onset time $T_{onset}$ of the index case [2] via:

$$T_{s}-T_{onset}+t_{p} \sim Weibull\left( 5.67,2.83 \right)$$

where $t_{p}>0$ is a constant chosen so that $p_{pre}=35\%$ of secondary infections occur prior to onset. Allowing for pre-symptomatic transmission in this way is realistic, but introduces the possibility that secondary infections could occur before infection of the index case. To prevent this, any randomly generated secondary infection times with $T_{s}<T_{onset}$ are discarded and another secondary infection time is generated instead. The population-wide generation time distribution is a model output (see Figure S1) and is consistent with empirical estimates [3, 4, 5].

- Symptomatic individuals who are not traced self-isolate after symptom onset. The time between symptom onset and isolation, $T_{iso}$, is Gamma distributed

$$T_{iso}\sim\Gamma\left( 0.62, 3.47 \right).$$

The distribution is found by fitting to the time between symptom onset and isolation in the New Zealand case data (see below) including all untraced individuals without a recent overseas arrival history and onset date between 8^th^ April and 8^th^ May 2020.

- All symptomatic cases get tested. i.e. $p_{test}=1$. In the absence of any contact tracing, the time, in days, between symptom onset and testing, $T_{test}$ is Gamma distributed

$$T_{test}\sim\Gamma\left( 1.22, 2.17 \right).$$

The distribution is found by fitting to the time between symptom onset and test date of the New Zealand case data (see below) of all untraced individuals without a recent overseas arrival history and onset date between 8^th^ April and 8^th^ May 2020.

- Isolation reduces infectiousness to $c_{iso}$ of initial infectiousness; this is assumed to take effect from either the isolation date or the test date, whichever is earlier.
- The time from testing to test result, $T_{result}$, (in days) is a minimum period of 0.5 days plus an exponentially distributed random variable with mean 0.5 days:

$$T_{result}-0.5 \sim Exp\left( 0.5 \right)$$

This is an approximate match to the New Zealand data (see below).

- A positive test result initiates tracing of the index case’s contacts. It is assumed a proportion $p_{trace}$ of contacts are successfully traced, though cases may be symptomatic, have isolated and even been tested by the time they are traced.
- The time between the index case’s test result and tracing of contacts, $T_{trace}$, is Gamma distributed with variance equal to 10% of the mean, i.e. tracing may be delayed but is completed quickly.
- Contacts traced before symptom onset go into partial isolation or quarantine, which reduces infectiousness to $c_{quar}$ of initial infectiousness. This is a weaker form of isolation during the pre-symptomatic stage ($c_{quar}\geq c_{iso}$).
- Once a traced contact develops symptoms, they are tested and fully isolated (infectiousness $c_{iso}$) immediately (i.e. there is no delay from onset to testing or isolation). After the test-to-result delay described above, a positive test result initiates tracing of the secondary case’s contacts.
- Contacts traced after symptom onset immediately go into isolation and are tested.
- Infections that remain asymptomatic do not get tested or isolated and their contacts are not traced.
- Asymptomatic infections can be traced contacts of an index case and be quarantined. However, they do not develop symptoms and so will not get tested or fully isolated.
- If isolation is not 100% effective tracing continues during isolation and contacts are still traced with probability $p_{trace}$.

**Stochastic branching process model.** The stochastic model for transmission of the virus is as follows:

- We segment the population into 3 age groups: 0-19 years, 19-65 years, over 65. The population is assumed to be well mixed within each group.
- The mean reproduction number $R_{sub}^{G}$ of asymptomatic individuals in any group $G$ was assumed to be 50% of the mean reproduction number $R_{clin}^{G}$ of eventually-symptomatic individuals in that group [6].
- We assume a moderate level of social distancing which reduces transmission rates to 70%.
- In the absence of case isolation measures, the expected number of secondary cases infected by individual $i$ is denoted $R_{i}$, referred to as the reproduction number for individual $i$.
- As well as population heterogeneity across the different groups, individual heterogeneity in reproduction numbers was included by setting $R_{i}=R_{clin}^{G}Y_{i}$ for eventually-symptomatic individuals in group $G$ and $R_{i}=R_{sub}^{G}Y_{i}$ for asymptomatic individuals in group $G$, where $Y_{i}$ is a gamma distributed random variable with mean 1 and variance 2. The number of secondary cases $N_{i}$ infected by individual $i$, in the absence of case isolation measures, is then generated according to $N_{i}\sim Poisson(R_{i})$. This is equivalent to assuming that the number of secondary cases infected by a randomly chosen eventually-symptomatic individual in group $G$, in the absence of case isolation measures, is $N_{i}\sim NegBin(R_{clin}^{G},k)$ with dispersion parameter $k=0.5$ [7].
- All individuals are assumed to be no longer infectious 30 days after being infected. This is an upper limit for computational convenience; in practice, individuals have very low infectiousness after about 14 days after symptom onset because of the shape of the generation time distribution (Fig. 1).
- The model is simulated using a time step of $\delta t=0.5$ days. At each step, infectious individual $i$ produces a Poisson distributed number of secondary infections with mean

$\lambda_{i}= R_{i}F(t,T_{trace,i}, T_{iso,i})\int_{t}^{t+\delta t} W\left( \tau-T_{onset,i} \right) d\tau$ (1)

where $W$ is the PDF of the Weibull distribution shown in Table 1, $R_{i}$ is individual $i$’s reproduction number, $T_{trace,i}$ is the time individual $i$ was traced (if applicable), $T_{iso,i}$ is the time individual $i$ was isolated (if applicable), and $F\left( t \right)$ is a function describing the reduction in infectiousness due to isolation:

$F\left( t,T_{trace},T_{iso} \right)=\left\{ \begin{matrix} 1 & t<T_{trace} \\ c_{quar} & T_{trace}\leq t<T_{iso} \\ c_{iso} & t\geq T_{iso} \end{matrix} \right.$ (2)

- The contact matrix $\Lambda$ gives the probability $\Lambda_{ij}$ that a secondary infection originating from group $i$ will be in group $j$, with $\sum_{j} \Lambda_{ij}=1$. New infections from group $i$ are distributed across groups according to these probabilities.
- The contact matrix assumes 50% of contacts are from the same age group. The remaining 50% are distributed across all three age groups in proportion to their relative size in the population, consistent with [8].
- The model was initialised with 10 seed cases infected at time $t=0$. $R_{eff}$ was calculated for each scenario using individuals who had experienced a full infectious period within 50 days of the seed cases.

**Performance indicators.** For each combination of parameter values investigated, we calculated the values of the four following performance indicators:

A. Proportion of secondary cases quarantined or isolated within 4 days of symptom onset in the index case.

B. Proportion of secondary cases quarantined or isolated within 4 days of quarantine or isolation of the index case.

C. Proportion of secondary cases with symptom onset within 4 days of symptom onset in the index case (i.e. serial interval less than 4 days).

D. Proportion of cases quarantined before symptom onset.

Time of quarantine or isolation means time of quarantine if there was a period of pre-symptomatic quarantine, or time of isolation otherwise. This is distinct from the time of diagnosis which may occur later for traced cases. These indicators were calculated in the model for secondary cases that eventually became symptomatic, all of whom have an onset time and an isolation time. Asymptomatic cases were not included in the metrics, despite being possible for some cases, as these are unlikely to be available in collected data. This includes cases who were not traced before symptom onset, and whose isolation date is therefore after their onset date. Indicators 1-3 additionally require the index case to be eventually-symptomatic. Values of these indicators were averaged over 1000 simulations for each combination of parameter values investigated.

**Data.** We used the New Zealand EpiSurv dataset, centrally managed by Environmental Science and Research (ESR) on behalf of the Ministry of Health (accessed 6 August 2020). We included the $N=101$ confirmed and probable cases with a symptom onset date between 8 April and 8 May 2020. Restricting to this period minimises the impact of changes that may have occurred as a result of expansion of the contact tracing system at the start of the epidemic in March and early April 2020. It also excludes the significant changes in behaviour and reduction in contact rates following the introduction of strict social distancing restrictions on 26 March 2020. We excluded cases with a recent international travel history ($N=8$) because the majority of these were infected overseas and/or were not identified as a result of contact tracing. All 93 included cases were symptomatic and had either an isolation or quarantine date and a symptom onset date recorded. 81 cases had at least one recorded index with recorded isolation, quarantine and onset dates, of these 37 had multiple potential index cases.

Case discovery is categorised in the dataset as either “contact of a case” ($N=81$), “sought healthcare” ($N=9)$ or “other” $(N=3)$, which could include surveillance testing. In New Zealand, during the period represented in the data, traced contacts were requested to quarantine at home but were not routinely tested unless they developed symptoms. This is consistent with the modelling assumptions. Of the 81 cases labelled as “contact of a case”, only 76 had a recorded index, the remaining 5 cases with a recorded index were all labelled as “sought healthcare”.

Defining traced contacts to be the 81 cases labelled as “contact of a case” implies a tracing rate of 87%. Of these cases, 57% were quarantined or isolated prior to symptom onset. There was almost no difference in the mean time from onset to testing between the traced and non-traced contacts (mean 2.8 days, standard deviation 2.6 days for sought healthcare; mean 3.0 days, standard deviation 2.3 days for contacts). This suggests that many of the cases labelled as “contact of a case” may have self-identified or been identified as a contact only after presenting to primary healthcare or testing services, as opposed to being traced by public health officials. This reinforces the view that directly quantifying the proportion of contacts who are traced is difficult and that this may not be accurately reflected in routinely collected public health data. Asymptomatic cases are not represented in the data as they were not routinely tested. However, spread from asymptomatic carriers is one source of transmission in the model.

**Empirical calculation of performance indicators.** We used the New Zealand EpiSurv dataset, centrally managed by ESR on behalf of the Ministry of Health (accessed 2 June 2020). We included the $N=95$ cases with no recent history of overseas travel and with a symptom onset date between 8 April and 8 May. Case discovery is categorised as either “contact of a case” ($N=82$), “sought healthcare” ($N=9)$ or other ($N=4)$. Of the 82 cases labelled as “contact of a case”, all were associated with at least one index case, allowing the values of the first three performance indicators defined above to be empirically calculated. For cases with multiple potential index cases, we calculated the following quantities

- Earliest time of onset in a potential index case $T_{onset}^{-}$.
- Latest time of onset in a potential index case $T_{onset}^{+}$.
- Earliest time of quarantine or isolation in a potential index case $T_{q}^{-}$.
- Latest time of quarantine or isolation in a potential index case $T_{q}^{+}$.

Using $T_{onset}^{+}$ and $T_{q}^{+}$ for all cases gave a minimum value for indicators 1-3. Using $T_{onset}^{-}$ and $T_{q}^{-}$ for all cases gave a maximum value for indicators 1-3. These minimum and maximum values corresponds to the two vertical dashed lines in Fig. 3A-C. Indicator 4 does not depend on the index case, so there is a unique value for this indicator (single vertical dashed line in Fig. 3D) which was calculated from all available case data.

**References**

1. James A, Plank MJ, Binny RN, Hannah K, Hendy SC, Lustig A, et al. A structured model for COVID-19 spread: modelling age and healthcare inequities. medRxiv. 2020.

2. Hellewell J, Abbott S, Gimma A, Bosse NI, Jarvis CI, Russell TW, et al. Feasibility of controlling COVID-19 outbreaks by isolation of cases and contacts. The Lancet Global Health. 2020.

3. Ferretti L, Wymant C, Kendall M, Zhao L, Nurtay A, Abeler-Dörner L, et al. Quantifying SARS-CoV-2 transmission suggests epidemic control with digital contact tracing. Science. 2020;368(6491).

4. Nishiura H, Linton NM, Akhmetzhanov AR. Serial interval of novel coronavirus (COVID-19) infections. International journal of infectious diseases. 2020.

5. Bi Q, Wu Y, Mei S, Ye C, Zou X, Zhang Z, et al. Epidemiology and transmission of COVID-19 in 391 cases and 1286 of their close contacts in Shenzhen, China: a retrospective cohort study. The Lancet Infectious Diseases. 2020.

6. Davies NG, Kucharski AJ, Eggo RM, Gimma A, Edmunds WJ, Jombart T, et al. Effects of non-pharmaceutical interventions on COVID-19 cases, deaths, and demand for hospital services in the UK: a modelling study. The Lancet Public Health. 2020;5:E375-E85. doi: 10.1016/S2468-2667(20)30133-X.

7. Lloyd-Smith JO, Schreiber SJ, Kopp PE, Getz WM. Superspreading and the effect of individual variation on disease emergence. Nature. 2005;438(7066):355-9.

8. Prem K, Cook AR, Jit M. Projecting social contact matrices in 152 countries using contact surveys and demographic data. PLoS computational biology. 2017;13(9):e1005697.
