# Supplementary figures and images for: Successful contact tracing systems for COVID-19 rely on effective quarantine and isolation
Source: PLoS One. 2021 Jun 3;16(6):e0252499. doi: 10.1371/journal.pone.0252499 (PMC8174731; doi:10.1371/journal.pone.0252499)

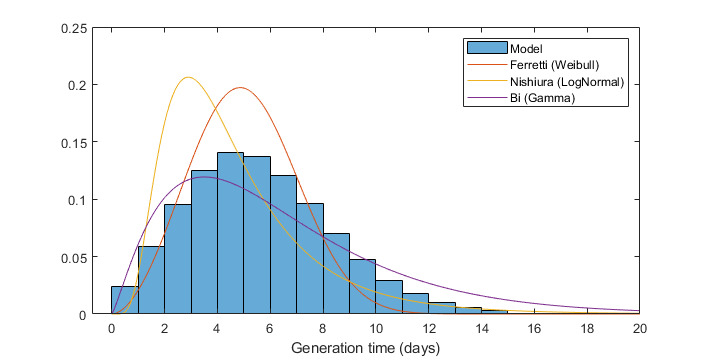

Supplement: S1 Fig — Three published generation time distributions are shown for comparison [2, 39, 40]. (TIF) [file pone.0252499.s002.tif]
